# Contribution to Surface Water Contamination Understanding by Pesticides and Pharmaceuticals, at a Watershed Scale

**DOI:** 10.3390/ijerph9124433

**Published:** 2012-12-04

**Authors:** Stéphanie Piel, Estelle Baurès, Olivier Thomas

**Affiliations:** 1 Environment and Health Research laboratory (LERES), EHESP School of Public Health, Avenue du Professeur Léon Bernard-CS 74312, Rennes Cedex 35043, France; E-Mails: stephanie.piel@ehesp.fr (S.P.); estelle.baures@ehesp.fr (E.B.); 2 Inserm, U 1085 Institute of Research in Environmental and Occupational Health (IRSET), Avenue du Professeur Léon Bernard-CS 74312, Rennes Cedex 35043, France; 3 SAUR Research and Development, 1 rue Antoine Lavoisier Saint Quentin en Yvelines 78064, France

**Keywords:** micropollutants, water quality, watershed, spatial variation, temporal variation

## Abstract

This study aims at understanding the presence of regulated and emerging micropollutants, particularly pesticides and pharmaceuticals, in surface water, regarding spatial and temporal influences at a watershed scale. The study of relations between micropollutants and other water quality and hydroclimatic parameters was carried out from a statistical analysis on historical and experimental data of different sampling sites from the main watershed of Brittany, western France. The outcomes point out the influence of urban and rural areas of the watershed as well as the impact of seasons on contamination variations. This work contributes to health risk assessment related to surface water contamination by micropollutants. This approach is particularly interesting in the case of agricultural watersheds such as the one studied, where more than 80% of surface water is used to produce drinking water.

## 1. Introduction

Among organic micropollutants monitored in water, pesticides are the most important class of hazardous substances. For example, in Europe, the Water Framework Directive (WFD; Directive 2000/60/EC) provides strategies against chemical pollution of surface waters and notably established provision for a list of Priority Substances (Annex X of the Directive) [1]. On the other hand the Drinking Water Directive (DWD) sets quality standards for drinking water quality at the tap (microbiological, chemical and organoleptic parameters) and the general obligation that drinking water must be wholesome and clean [2]. World Health Organization (WHO) guidelines are used as a basis for the standards in the WFD and DWD [3], and precise that “pesticides” means insecticide, herbicide, fungicide, nematicides, acaricide, algicide, rodenticide and organic slimicide substances and related products (including growth regulators), their metabolites, their degradation or relevant reaction products. Two quality limits have been set in water intended for human consumption: 0.10 µg/L for each substance (except four of them: aldrin, dieldrin, heptachlor and heptachlor epoxide, for which the applicable limit is 0.03 µg/L, which corresponds to the WHO guideline value) and 0.50 µg/L for total pesticides quantified.

In the United States, the Clean Water Act (USEPA) is the cornerstone of surface water quality protection [4]. The statute employs a variety of regulatory and non-regulatory tools to reduce direct pollutant discharges into waterways, finance municipal wastewater treatment facilities and manage polluted runoff. These tools are employed to achieve the broader goal of restoring and maintaining the chemical, physical and biological integrity in the nation’s waters. Secondly, the Safe Drinking Water Act (USEPA) is the main federal law that ensures the quality of drinking water [5]. Under SDWA, EPA sets standards and oversees the states, localities and water suppliers who implement them. National Primary Drinking Water Regulations (NPDWRs or primary standards) are legally enforceable standards that apply to public water systems. Primary standards protect public health by limiting the levels of contaminants in drinking water, like some pesticides.

The presence of pharmaceuticals in surface and groundwater resources available for human consumption is a current worldwide public health issue. No regulation on the monitoring of these substances and therefore quality standards for the resource or treated water exist today in Europe. A group of experts was formed in 2009 and commissioned by the WHO to review the available scientific literature in order to identify key issues related to the health risk of human exposure to pharmaceutical residues present in trace amounts in water, to judge the potential contributions of changes of current regulations on drinking water quality and to provide necessary recommendations [6]. Their conclusion is that health risk has not been yet demonstrated. WHO emphasizes in its report the lack of sufficient knowledge about the health risks associated with chronic exposure to low levels of pharmaceutical residues present in water as mixtures. Therefore, the WHO urges the scientific community to further research this topic in order to assess the effects related to multiexposition of these residues (synergistic and additive effects). Very recently, the European Commission decides to propose the introduction of four pharmaceuticals (ibuprofen, diclofenac, 17α-ethinyl estradiol, β-estradiol) in the list of priority substances annexed to the WFD. In the United States also, some pharmaceuticals are on the Third Contaminant Candidate List (CCL3) in order to evaluate if national drinking water regulations are needed to protect public health.

In this context, the aim of the present study is to contribute to a better understanding of the contamination of surface waters by some micropollutants (pesticides and pharmaceuticals) at a watershed scale. More precisely relationships between micropollutants with basic water quality and hydroclimatic parameters will be studied from historical and recent experimental data. Seasonal and spatial variations in relation with land use and agricultural practices will also be considered.

## 2. Material and Method

### 2.1. Field Characteristics

This study was carried out in Brittany, which is the premier agricultural region of France, especially in terms of animal farming for milk and meat, corn cultivation, and vegetable crops. Its main activity is the food industry, which accounts for 80% of the French production [7]. Surface water accounts for 80% of the drinking water resource available in the watershed [8]. The biggest watershed in Brittany is the Vilaine basin, which covers two thirds of the region (10,500 km²). The main river the Vilaine, which is about 220 km in length from its source to its mouth and crosses Rennes, a city of approximately 300,000 inhabitants. Furthermore located at the extreme downstream of the basin is the largest drinking water treatment plant (DWTP) of the region, with a nominal production capacity of 100,000 m^3^ per day corresponding to more than 1 million inhabitants connected in summer.

The two sub-watersheds, the Meu and Oust, are predominantly under agricultural pressure. Table 1 gives some characteristics of these two river basins. On the Meu area, agriculture is focused essentially on mixed farming and stockbreeding and some intensive agricultural production areas exist. On the other side the upstream part of the Oust basin has an important food industry activity. The median part of the Oust sub-watershed is mainly oriented towards stockbreeding—65% of farms produce milk whereas enclosed breeding (poultry, pig, rabbit) represent approximately 22% of holdings. Soilless cultures are spread uniformly throughout the whole basin. Finally on the downstream part of the Oust sub-watershed, agriculture is predominantly dairy, but poultry and pig farming are also well represented.

**Table 1:** Characteristics of the main sub watersheds of the Vilaine.

Characteristics	Meu	Oust
Length (km)	87	147
River basin area (km²)	815	3,614
Number of agricultural holdings	1,300	1,789
Utilised agricultural land (ha)	54,000	68,280

### 2.2. Historical Data Set

**Figure 1:**
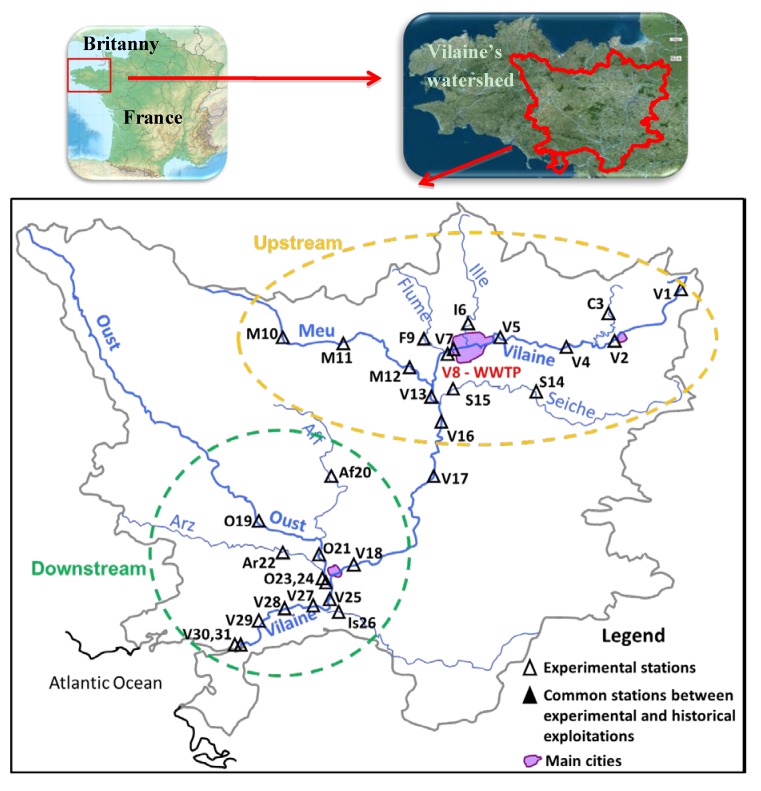
Location of stations.

Historical data are provided from the Osur Web (Water Agency “Loire-Bretagne”) database for water quality [9], and from the Banque Hydro (Ministry of Ecology) database for the river flows (Q) measured at the same sites [10] (Figure 1). Seven sites have been chosen because of the number of data on pesticides concentrations as well as their strategic location on the main basin, the Vilaine and on the two main sub-watersheds, the Meu and Oust. They have also been selected for experimental campaigns (see hereafter). Among these seven stations, three are located in the upstream part of the Vilaine basin (V1, V5 and M12), three in the downstream part (V18, O19 and V25), and one downstream the main wastewater treatment plant (WWTP), V8, designed for 360,000 inhabitants equivalent (Rennes). Data acquisition periods are different considering the stations’ histories: from 1997 to 2010 for V5, V18, O19 and V25; from 2002 to 2010 for V1; from 2002 to 2009 for M10 and from 1997 to 2006 for V8.

In addition, daily precipitation rates have been collected from the Meteo France database [11]. Among the historical chronicles available, two specific years have been selected, 2002 and 2003, corresponding to rainy and dry years, respectively. Characteristic temperatures and precipitation rates are presented in Table 2. The year 2002 presents the highest percentile 90 of daily precipitation rate of all the data acquisition years (from 1997 to 2010) and the year 2003 presents the highest percentile 90 of temperature and the lowest mean and percentile 90 daily precipitation rate.

**Table 2:** Characteristic temperatures and precipitation rates of historical data sets.

	Mean Temperature (°C)	Percentile 10 Temperature (°C)	Percentile 90 Temperature (°C)	Mean Daily Precipitation Rate (mm/day)	Percentile 90 Daily Precipitation Rate (mm/day)
2002 (rainy)	13.04	7.54	18.96	2.75	8.42
2003 (dry)	13.20	4.58	21.56	1.68	5.72

**Table 3:** Pesticides of interest, their usage and quality standards.

Pesticides	Nature	Usage	European environmental quality standards (µg/L)	European drinking water standards(µg/L)	US drinking water quality standards (µg/L)
Atrazine * (AT)	Corn herbicide	Agricultural	0.6	Individual substance 0.1 Total pesticides 0.5	3
Desethyl atrazine (ATdes)	Atrazine metabolites	-	No data		No data
2-hydroxy-atrazine (2HAT)					
Glyphosate (GLYP)	Total herbicide	All users			70
AMPA	Glyphosate metabolite	-			No data
Diuron (DIU)	Total herbicide	Individuals, local authorities	0.2		-
Isoproturon (ISOP)	Cereal herbicide	Agricultural	0.3		No data
Mecoprop (MECOP)	Corn herbicide	Agricultural	No data		
Trichlopyr (TRIC)	Total herbicide	All users			

* Prohibited in France in 2003.

Concerning water quality, physicochemical parameters have been considered (NH_4_^+^: ammonia, KN: Kjeldhal nitrogen, NO_3_^−^: nitrate, “PO_4_”: orthophosphate, Pt: total phosphorus, TOC: total organic carbon, DOC: dissolved organic carbon, TSS: total suspended solid, Turbi: turbidity, ChlA: chlorophyll A, O_2_S: Oxygen saturation rate, Cond: conductivity) as well as pesticides, from OSUR Web data base. Numerous pesticides were analyzed but, hopefully, many were detected below quantification limits. For the significance of statistical analysis, only those detected above the quantification limit with a frequency above or equal to 20% have been retained. It could be underlined these molecules are only herbicides. Table 3 summarizes the pesticides of interest and presents their different usage. It should be precised that no analyses of pharmaceuticals were available.

### 2.3. Experimental Data Set

**Figure 2:**
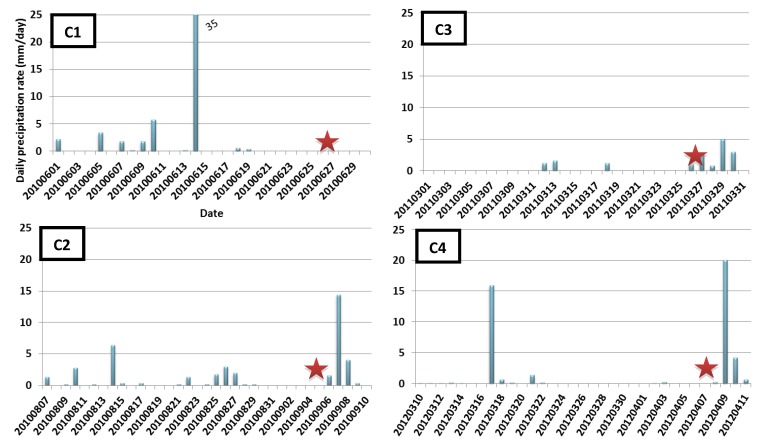
Daily precipitation rate of the four sampling campaigns (experimental datasets); 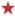: correspond to the sampling dates.

Four sampling campaigns have been carried out between 2009 and 2012 on the Vilaine and its tributaries at 31 sampling stations (Figure 1), three during dry periods (C1, C2 and C3) and one after a rainfall event (C4). A sampling campaign was considered as rainy for a rainfall height of 10 mm minimum in 24 h before sampling. Daily precipitation rates are presented on Figure 2.

Among the 31 samples, 19 were collected from a bridge using a bucket, 11 from the bank using a pole according to the AFNOR standards (FD T90-523-1, February 2008), and the last one directly sampled in the chlorination tank of the DWTP. In the same time, *in situ* measurements of a variety of parameters (pH, temperature, turbidity, conductivity, dissolved oxygen concentration, oxygen saturation rate and oxidation/reduction potential) were also realized. In addition, appropriate flasks were used according to the type of analysis realized in the laboratory, for instance brown bottles for micropollutants to avoid photodegradation, polyethylene flasks with hydrochloric acid for TOC in order to conserve the sample, *etc*. Samples were conserved at 5 °C ± 3 °C during the transport.

Basic physicochemical parameters (the same as for historical data), 65 pesticides (triazines, phenyl urea, triazoles, nitrophenols, chloroacetamides, phenoxy carboxylic acids…), 12 human pharmaceuticals (HPs) and 10 veterinary pharmaceuticals (VPs) have been analyzed on each station by liquid chromatography coupled with mass tandem spectrometry. In order to compare with historical data set, the same nine pesticides have been studied in a statistical analysis. Among the most frequently quantified HPs and VPs, five HPs and one VP have been selected in experimental datasets for statistical analysis: caffeine (CAF, psychostimulant), carbamazepine (CBZ, anticonvulsant), sulfamethoxazole (SFX, antibiotic), oxazepam (OZP, anxiolytic), iopromide (IOP, ionated contrast media) and sulfamethazine (SFZ: veterinary antibiotic). All parameters were measured and analyzed with respect to standardized methods (ISO/AFNOR) such as NF EN ISO 11369 (1997) for pesticides [12,13].

In addition, river flows have been collected from the Banque Hydro data base on each sampling stations. Considering the area of the field experiment (watershed) with more than 200 km between the two extreme sampling stations, the duration of one sampling campaign was at least 2 full days. This experimental time period did not guarantee constant weather conditions, as for example for C2 following a dry period, but carried out in rainy conditions for some sampling stations.

### 2.4. Statistical Exploitation

#### 2.4.1. Principal Component Analysis

Principal Component Analysis (PCA) was performed using the R 2.11.0 software (package “FactoMineR”). PCA is a powerful pattern recognition technique that explains the variance of a large dataset of intercorrelated variables, the water quality parameters in this study, with a smaller set of independent variables, the principal components [14]. It helps to extract and identify the factors/sources responsible for variations of river water quality at the different sampling sites. Results are presented in variables factor maps (VFMs) form. The contribution of all parameters is used for the construction of each dimension of the PCA. This construction allows detecting among them which ones are extreme and the most responsible for the water quality variations [15]. VFMs also allow observation of correlation between parameters. For each VFM, only two dimensions have been considered in the interpretation because of their relative weight in variance explanation. PCAs have been realized on each campaign data set and on 2002 and 2003 historical data sets corresponding respectively to a rainy year and a dry year. It has to be underlined that values below quantification limit are replaced by the quantification limit divided by two in historical and experimental databases. Finally, these analyses allow studying hydroclimatic impacts on micropollutants and relation between micropollutants and other water quality parameters.

#### 2.4.2. Hierarchical Clustering on Principal Components (HCPC)

The objective of classification is to divide the sample into groups of homogeneous observations, each group being clearly differentiated from the others. Such a hierarchy could be summarized by a hierarchical tree, called dendrogram, whose nodes symbolize the various subdivisions of samples. Elements of these subdivisions are objects placed at the lower end of their branches. Node levels indicate the degree of similarity between the corresponding objects, the more the node is down the more objects are similar [16]. In this study, the hierarchical classification aims at classifying sampling stations according to their water quality. It is called “principal component” as hierarchical clustering is performed following a PCA of the different databases. Indeed for this study, PCA scores have been used to realize the HCPC analysis. This analysis was performed using the software R 2.11.0 (package “FactoMineR”) on historical and experimental data. Finally, these analyses allow identifying temporal (seasonal variation) and spatial impacts (from rural or urban area) on the presence of characteristic micropollutants.

## 3. Results

### 3.1. Evolution of Pesticides

**Figure 3:**
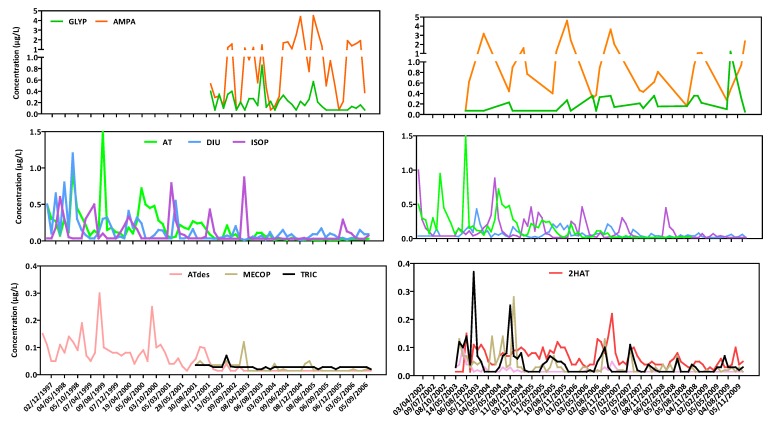
Evolution of pesticides on V8 (**left**) and M12 (**right**) (historical data sets).

Figure 3 presents the evolution of pesticides on V8 (urban area) and M12 (agricultural area) according to historical data sets. Three scales of pesticides concentration have been highlighted considering the order of magnitude of maximum concentrations of each molecule: around 5 µg/L for AMPA (its parent compound, GLYP, is presented on the same graph); around 1–1.5 µg/L for AT, DIU and ISOP and below 0.4 µg/L for ATdes, 2HAT, TRIC and MECOP.

Concentrations of AMPA are clearly higher than its parent compound, GLYP, but each AMPA concentration peak coincides with a GLYP peak. The use of this type of pesticides seems to be constant in time, from 2003 to 2010. On the other hand, AT presents some high concentration peaks above 0.5 µg/L until 2001 for V8 and until 2004 for M12 and then concentrations decrease considerably below 0.1 µg/L. This observation could be explained by its prohibition in 2003 in France. Its metabolite (ATdes) concentration follows the same trend, whereas 2HAT seems to present a constant concentration from 2003 to 2010 at M12. ATdes is formed by microorganism degradation in soils and 2HAT by hydrolysis and photolysis of AT and ATdes in water. Thus the constant presence of 2HAT could be due to the persistence of AT and ATdes in soils time of disappearance for half of the chemical (DT_50_ = 75 days) and of their rapid photolysis in water (DT_50_ = 2.6 days) in the 2HAT (Pesticides Properties Data Base, http://sitem.herts.ac.uk/aeru/footprint/en/).

Concerning DIU, concentration peaks are less specific to a time period and its use seems to decrease since 2001 with concentration peaks below 0.2 µg/L. In addition, concentrations are lower for the agricultural station M12 and the use clearly decreases, considering its quantification below 0.1 µg/L since 2007. On the other side, concentration peaks of ISOP are regularly quantified at the beginning of the year, especially in March, periods which follow the period of the pesticides’ use on the fields and the rainy period (winter). Since 2007 ISOP continues to be detected but at relatively low levels.

TRIC is rarely detected on V8 (urban) but more frequently at M12 (rural), with concentration peaks up to 0.36 µg/L. After 2004, concentration peaks decreased below 0.1 µg/L, but TRIC continued to be regularly detected. Its concentration in water could be lower than the other pesticides because of its known quick hydrolysis and photolysis in water (DT_50_ = 8.7 and 0.1 days respectively). Finally, the same observations could be drawn for MECOP and could be explained by its quick biodegradation in soils (DT_50_ = 8.2 days).

### 3.2. Relation between Micropollutants and Other Parameters

The most commonly applied multivariate method in watershed studies is PCA [17]. This literature survey reviews 49 published papers on this subject. All studies present the results of PCA applied to data of specific environmental factors, processes, and/or contamination sources but any of them include data on pesticides or pharmaceuticals concentrations like in our study.

**Figure 4:**
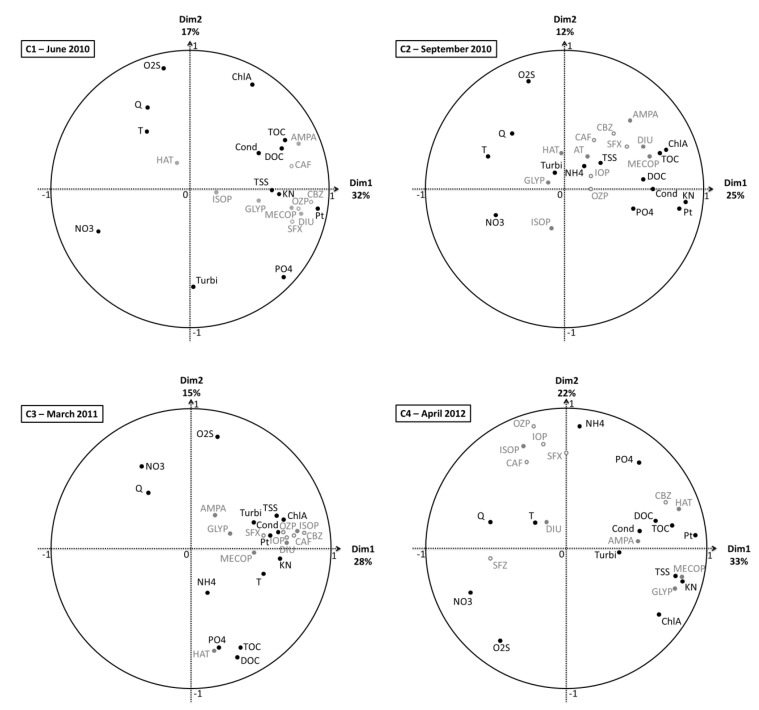
Results of the PCA of the four campaigns (experimental datasets); physicochemical and hydroclimatic parameters are in black and micropollutants in grey. (NH_4_: ammonia, KN: Kjeldhal nitrogen, NO_3_: nitrate, PO_4_: orthophosphate, Pt: total phosphorus, TOC: total organic carbon, DOC: dissolved organic carbon, TSS: total suspended solid, Turbi: turbidity, ChlA: chlorophyll A, O_2_S: Oxygen saturation rate, Cond: conductivity, Q: daily flow, T: temperature).

Figure 4 presents the VFMs of each sampling campaign. In general, for all campaigns, dimension 1 (Dim1) is linked to nutrients and organic loads (TOC, KN and/or Pt…), which represent a pollution gradient [18], whereas a slight difference appears with regard to flow rate Q, since it is closer to dimension 2 (Dim2) for C1, C2 and C3 than for C4, where it is linked to Dim1, probably due to the rainfall events of 20 mm/day.

For C1 and C3, all pesticides are grouped and linked to Dim1 and thus correlated to nutrients and organic loads. But during C2 and C4, some pesticides are associated to hydroclimatic factors, ISOP and GLYP for C2 and DIU and ISOP for C4. This observation is likely in relation to the impact of leaching and runoff during and after rainfall events, respectively for C2 during which it was raining and C4 after a rainfall events.

Concerning human pharmaceuticals distribution, points on VFMs are relatively close, which can be explained by identical correlation with Cond, TOC, DOC, TSS, KN and Pt for C1, C2 and C3. For C4, only CBZ is always correlated with the previous parameters whereas OZP, IOP, CAF and SFX move closer to Q and T. In addition, during dry campaigns veterinary pharmaceuticals were quantified at low frequencies (20% of samples) and at low concentrations, between 8 and 15 ng/L, as observed by Veach *et al*. [19]. Moreover SFZ was more often quantified, around 50%, at concentrations up to 50 ng/L for C4 after rainfall events of approximately 20 mm/day. Finally, SFZ is clearly correlated with Q and NO_3_, always for C4.

In a previous study, Piel *et al*. identified groups of sampling stations from historical data sets on this watershed, using the same groups of parameters (pollution gradient, hydroclimatic, leaching and runoff), except micropollutants [18]. In the present study, micropollutants are correlated to these groups and the PCA on each campaign allow identifying differences among relationships between micropollutants and parameters depending on the period of the year and thus on different climatic conditions. Therefore, the watershed showed temporal and spatial variations which will be developed hereafter.

### 3.3. Influence of Urban and Rural Area

**Figure 5:**
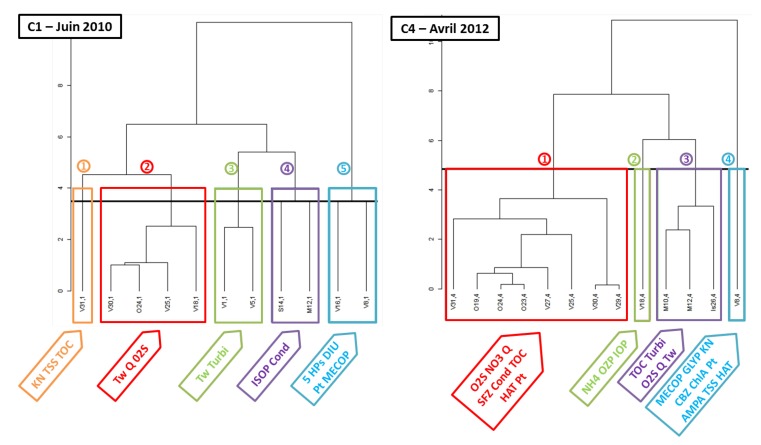
Results of HCPC during C1 and C4.

Figure 5 shows dendograms obtained with HCPC analyzes with experimental data sets, C1 and C4, respectively, carried out during dry and rainy periods. In addition, the most significant water quality parameters (*p* > 0.05) related to each cluster are precised under each of them.

During both campaigns, clusters numbered from 1 to 5 for C1 and from 1 to 4 for C4, are clearly identified regarding their location on the watershed. A previous study using PCA for physicochemical data exploitation showed the same trend of spatial classification, with the “upstream group” rather dominated by circumstantial effects like rainfall events, the “downstream group”, dominated by chronic effects with continuous discharge, and the “discharge” group [18].

In the present study, a slight difference occurs with the apparition of a new class regrouping the two agricultural sub-watersheds, the Meu and Seiche for C1, and the Meu and Isac for C4. During C1, they are associated to the presence of pesticide ISOP and Cond and during C4, they appear to be clearly impacted by the rainfall event of 20 mm/day linked to four characteristic parameters: O_2_S, TOC, Turbi and Q. Indeed, relation linking these three parameters is well known, with an increase of flow, turbidity and total organic carbon concentration after heavy rainfall events [20]. In addition, during both campaigns, V8 located downstream a WWTP and corresponding to the “discharge group”, appears in the cluster defining by KN, Pt and most of the micropollutants. In addition, during a dry campaign (C1), V16 located downstream of Rennes and the confluence with two important tributaries, is in the same cluster. The impact of the WWTP seems to persist until this station.

A few studies using cluster analysis found a gradient of water quality group, from “less polluted” to “high polluted” [21,22,23,24,25]. Here, the gradient begins with cluster 1 corresponding to the “less polluted” to cluster 5 for C1 and cluster 4 for C4, corresponding to the “highest polluted”. The definition of these extreme groups is coherent because V31 corresponds to drinking water, V8 to the station downstream the WWTP and V16 to the stations downstream of Rennes and the Meu sub-watershed. Besides, water quality seems to be improved along the river since downstream stations are in the cluster 2.

### 3.4. Seasonal Variation of Micropollutants Contamination

The seasonal variation of pesticides and pharmaceuticals could not be studied with the same approach depending on the molecule type and on the station location. Human pharmaceuticals consumed during all the year are thus continuously infused into the river via WWTP effluents [26] whereas pesticides are used only during certain authorized periods, especially in agricultural areas. 

**Figure 6:**
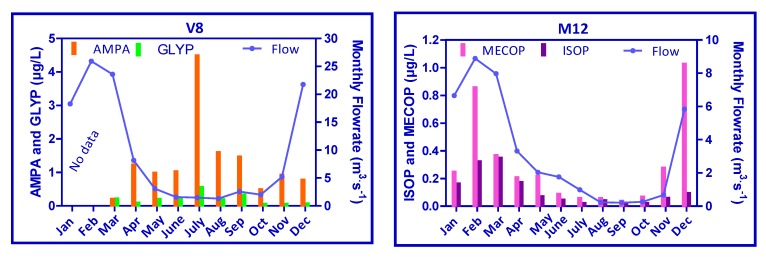
Evolution of monthly flow rate and monthly concentrations of AMPA, GLYP, MECOP and ISOP on urban and rural areas (historical data).

Besides, according to historical data, two pesticides profiles have been found regarding two specific stations of the Vilaine’s basin, the first in an urban area (V8) and the second in a rural one (M12) (Figure 6).

GLYP is a general herbicide used in agricultural and non-agricultural areas. This was confirmed by its quantification frequency oat stations V8 and M12, with 68% and 57% of occurrence (data above the quantification limit), respectively. The evolution of concentrations of GLYP and AMPA, its main metabolite, followed the same trend, which is the opposite of the flow rate one (Figure 6). This phenomenon seemed to be governed by dilution effects. Moreover AMPA concentrations are clearly greater than GLYP ones and exceed the French quality standards concentrations of 0.1 µg/L for individual molecules for drinking water resources. In addition, V8 is located downstream of a WWTP, so it appears either that treatments are not really effective against this type of molecules, or either that this type of molecules are formed during the actual treatment steps. Besides, considering both molecules, AMPA and GLYP, in a rural area (M12), a same yearly trend could be observed with a maximum concentration (2 µg/L) lower than in urban area (data not shown here).

MECOP and ISOP are herbicides used in agricultural areas for agricultural crops. This was confirmed by the detection frequency at stations V8 and M12, with 23 and 56% of samples for MECOP, and 38 and 65% for ISOP, respectively. Contrary to GLYP and AMPA, the evolution of MECOP and ISOP concentrations follows the same trend as flow rate. These observations seem to reflect the impacts of leaching and runoff. Moreover high concentrations levels during winter highlight the fact that during the authorized period of MECOP and ISOP use on wheat, barley and rye, leaching and runoff are intensified. In addition, a similar trend could be observed in an urban area (V8) for MECOP and ISOP, with a maximum concentration of 0.45 µg/L (data not shown here).

Despite this difference, pesticide inputs from urban areas (maximum 4.5 µg/L) are at least more important as those from agricultural areas (maximum 1.1 µg/L) whereas historically, agricultural pesticides have received substantially more attention than biocidal compounds from urban use, despite being used in similar quantities [27].

**Figure 7:**
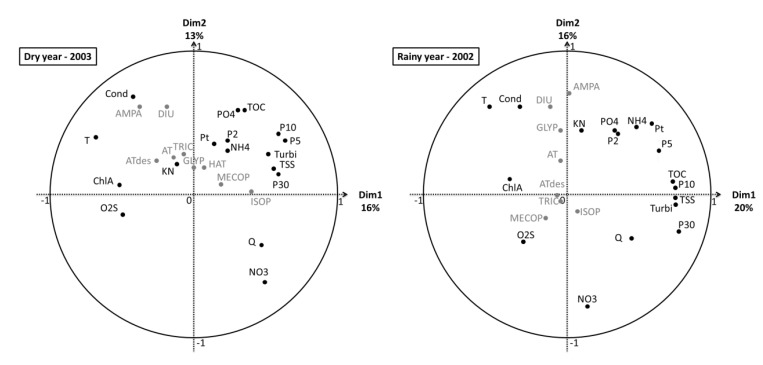
PCA results on dry and rainy years.

Subsequently, in order to compare dry and rainy conditions, PCA has been carried out on the 2002 (rainy) and 2003 (dry) historical data sets. Figure 7 presents their respective VFMs.

During the rainy year, all pesticides are correlated with hydroclimatic parameters (NO_3_, Q, Cond, T). However, substances mainly used in agriculture, MECOP and ISOP, are correlated positively with Q, NO_3_ and O_2_S due to leaching and runoff, whereas those of general use are negatively correlated due to dilution phenomena. On the VFMs of the dry year, both agricultural molecules are correlated to Q, Turbi, TSS and precipitation rates whereas others are linked to organic load (NK, Pt, PO_4_, TOC and NH_4_) and always negatively correlated to Q and NO_3_, both due to the same explanation.

However, the sum of variance of these two analyses for the two first dimensions is relatively low, 29 and 36%, respectively, probably due to a mixture effect of all seasons. Therefore a conclusion on the impact of the climatic trend of the year is difficult to draw. To go further, a HCPC analysis was carried out to point out more precisely the seasonal impact on resource quality, especially regarding pesticides. Notably because weather events, weather patterns and seasonal variations are the first causes of danger to the resource [28].

**Figure 8:**
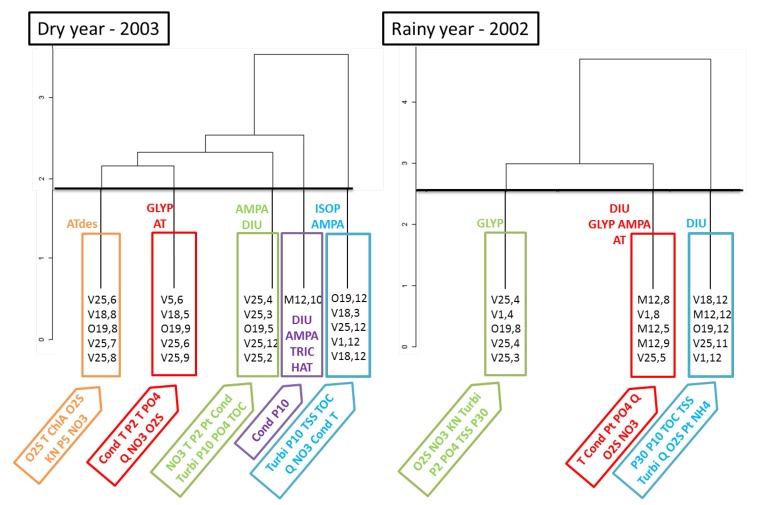
HCPC results on dry and rainy years.

Figure 8 shows the two dendograms obtained with HCPC analyzes with historical data sets of dry and rainy years. Indexes precised after the name of the station correspond to the month’s number, for instance 8 for August. Only stations representative of each cluster are presented on the graph. In addition, water quality parameters defining each cluster are precised on the figure.

Two clear observations appear: (i) the majority of the stations are those located in the downstream part of the basin during both years; in addition, during rainy year the station on the sub-watershed the Meu appears more frequently (ii) clusters seem to be formed regarding seasons or/and hydroclimatic conditions. During dry years, the first cluster clearly corresponded to summer ,with the highest T, low O_2_S and some localized rainfalls. Cluster 2 was constituted by months before and after summer (May, June and September) characterized by the lowest flow rates, low O_2_S and rare rainfalls. Then the three other clusters, 3, 4 and 5 were clearly those impacted by rainfalls. Cluster 3 was a mixture of spring and winter and presents nutrients and organic load parameters as significant parameters, PO_4_, Pt and TOC. Whereas cluster 5 was clearly impacted by more intensive rainfalls which occur particularly during winter and sometimes in Spring, with significant parameters corresponding to Turbi, TSS, TOC, Cond and NO_3_ known to be linked with this kind of events [20]. Then the last cluster, 4, is constituted by a single station, M12, which is a tributary quickly impacted by the first rainfalls following summer and which has the highest conductivity values of the watershed, especially during rainy periods.

During the rainy year, only three clusters have been identified. Cluster 1 was predominantly constituted by Spring months impacted by regular rainfalls and the presence of nutrients. Cluster 2 corresponded to the lowest flow rate periods, Summer (August) and before/after Summer (May and September), characterized by the same parameters than the cluster 2 of the dry year. Finally cluster 3 was clearly impacted by intensive rainfall events during Winter period (November and December) and for which significant parameters are relatively similar to cluster 5 of the dry year.

Whatever the trend of the year, dry or rainy, seasons really impact resource quality because they correspond to different hydroclimatic conditions but also to different types of land use or pesticide use practices. At least one pesticide appears in each cluster. Urban pesticides appear more frequently than agricultural ones, which could be explained by the fact that urban uses of herbicides exceed agricultural uses, and that transfer coefficients were also higher in urban areas [29]. Therefore, GLYP and DIU are the most used product in urban areas and are three times present in cluster 3 of dry and rainy years and in cluster 2 of the rainy year, probably due to their use on impervious surfaces [29]. Besides, the main agricultural pesticide of this study, ISOP, appears only in a Winter cluster (cluster 5) during the dry year, corresponding to the authorization period of this substance and rainfall events period.

## 4. Discussion

The first point to be discussed is a critical analysis of the methodology including historical and experimental water quality data exploitation on a given watershed. Regarding the choice of the studied watershed, it has to be underlined that this one is located in a specific area with a predominant agricultural pressure and other studies should be pursued on other river basins of different characteristics in order to improve the significance of the outcomes.

In addition, historical data sets present some heterogeneous series because sampling frequency is variable and depending on the monitored station. Depending on the program the list of substances monitored can vary from one sampling site to another and in some cases may be low for some. Experimental data sets are series collected during specific times selected by weather conditions, including dry or rainfall periods, and consequently are representative of rather “extreme” conditions. On the other hand, the number of campaigns of water quality parameters could be completed to gain in relevance. Considering these observations, the main outcomes of this study must be shown as trends on water quality variability in a context of intensive agricultural area. 

Many studies of water quality include molecule screenings giving concentration ranges of micropollutants in surface water [30,31,32,33]. Besides the knowledge of the occurrence and fate of these substances, it is also important to understand how and why these molecules are present and their concentrations vary, namely because of their potential adverse health effects such as those well known for pesticides. Depending on the type of pesticide (USEPA), some molecules such as organophosphates and carbamates, affect the nervous system; others may be carcinogens or may affect the hormone or endocrine systems in the body [34]. Concerning pharmaceuticals, toxicity data are sparsely published. Few studies deal with the risk of the presence of hormones in the water intended for human consumption and data indicate that it is not without risk to human health [35]. Another interest to consider the variability of a wide variety of micropollutants in resources and drinking water is that risks are often assessed only on the base of individual compounds. The potential effects of a mixture of pharmaceuticals on human health, at short or long term, are not yet known [33]. Additive or synergistic/antagonistic effects of the micropollutants are to be expected in accordance to what has been observed in ecotoxicology on aquatic organisms in contact with mixtures of organic contaminants [36,37]. In such complex mixtures, chemical and biological interactions may occur, which cannot be easily extrapolated from the knowledge of structure and reactivity of individual compounds.

In addition, another current issue is pointed out with climate change impact. Bloomfield *et al*. deal with the impact of climate on the presence of pesticides [38]. The main climate drivers for changing pesticide fate and behavior are changing rainfall patterns (changes in seasonality and intensity) and increased temperatures. In the long term, land-use change driven by changes in climate may have a more significant effect on pesticides in the environment than the direct impacts of climate change on specific pesticide fate and transport processes [38].

Finally, one key issue of our study should have been to be able to determine as precisely as possible which families of micropollutants and which substances alone or in mixture could be found in waters depending on watershed characteristics, land use, environmental and hydroclimatic conditions. Unfortunately, even if our results provide useful information for some investigated micropollutants likely to be detected in surface waters, for comparable watersheds, a lot of efforts are required before proposing relevant modeling with practical outcomes in terms of health risks management. Starting from the results of the present study, further investigation should lead to a first validation concerning water contamination by pesticides and pharmaceuticals, according to their relations with spatial and temporal factors. The final objective could thus be a tentative to predict the type of micropollutants mixture present in water resources at specific spatial and temporal conditions, using only weather forecasts and easily analyzable water quality parameters. These predictions could notably be used in the frame of small water services management particularly vulnerable to water quality degradation. 

## 5. Conclusions

This study must be considered as a contribution to the understanding of the contamination of surface water by micropollutants by focusing on spatial and temporal influences on variations in pesticide and pharmaceutical concentrations in a given agricultural watershed. In addition, some relations between water quality and hydroclimatic parameters and micropollutants have been pointed out using PCA on experimental and historical data sets. Micropollutants are essentially linked to nutrients and organic load, except during and after rainfall events where some pesticides and pharmaceuticals are linked to hydroclimatic factors, respectively, because of leaching and runoff or dilution effects. The cluster analysis on experimental data allows identifying a gradient of water quality groups, from less polluted, downstream stations, to more polluted stations, downstream of a WWTP. Then the cluster analysis on historical data sets shows that seasonal and hydroclimactic conditions really impact the resource quality.

Obviously these results depend on the geological substrate of the basin, on the hydrology and morphology of the watershed, on the land use, *etc*, and thus could not be generalized or modeled without further investigation on other type of watershed. Finally, this work should be pursued for a better characterization of hydroclimatic based relationships between micropollutants and other water quality parameters for improving health risk assessment.
